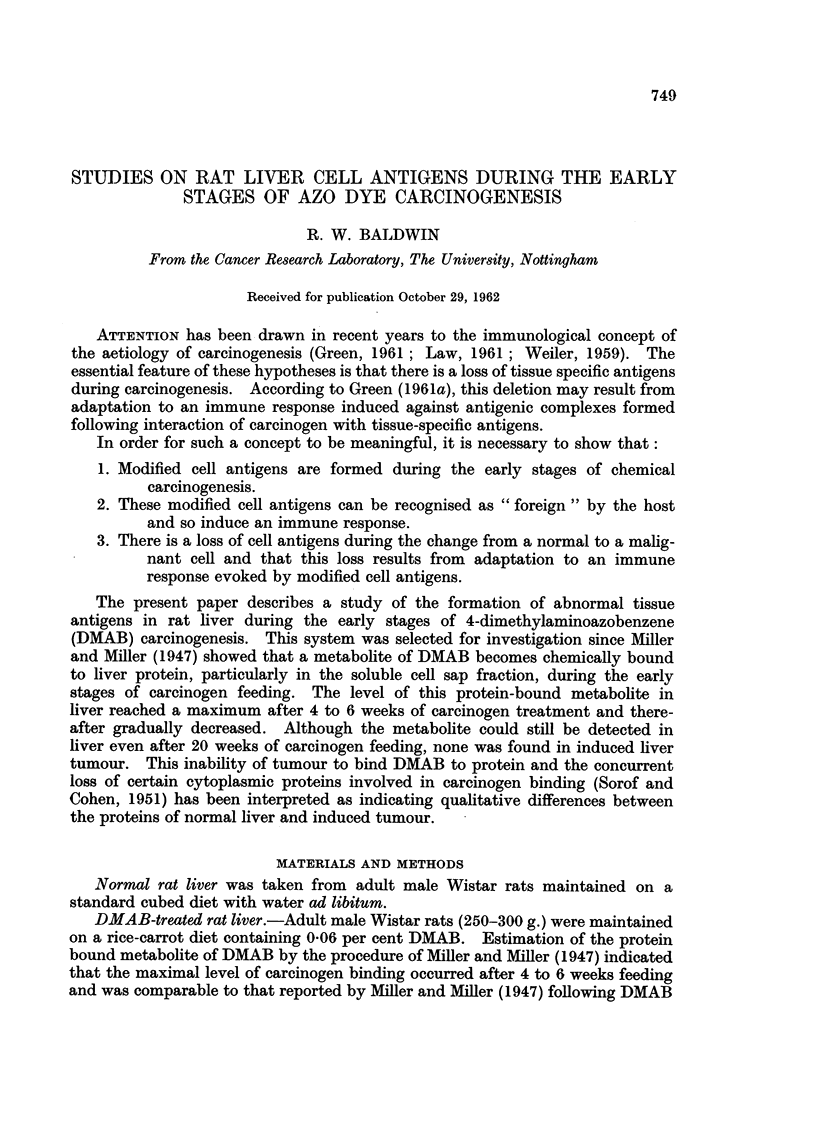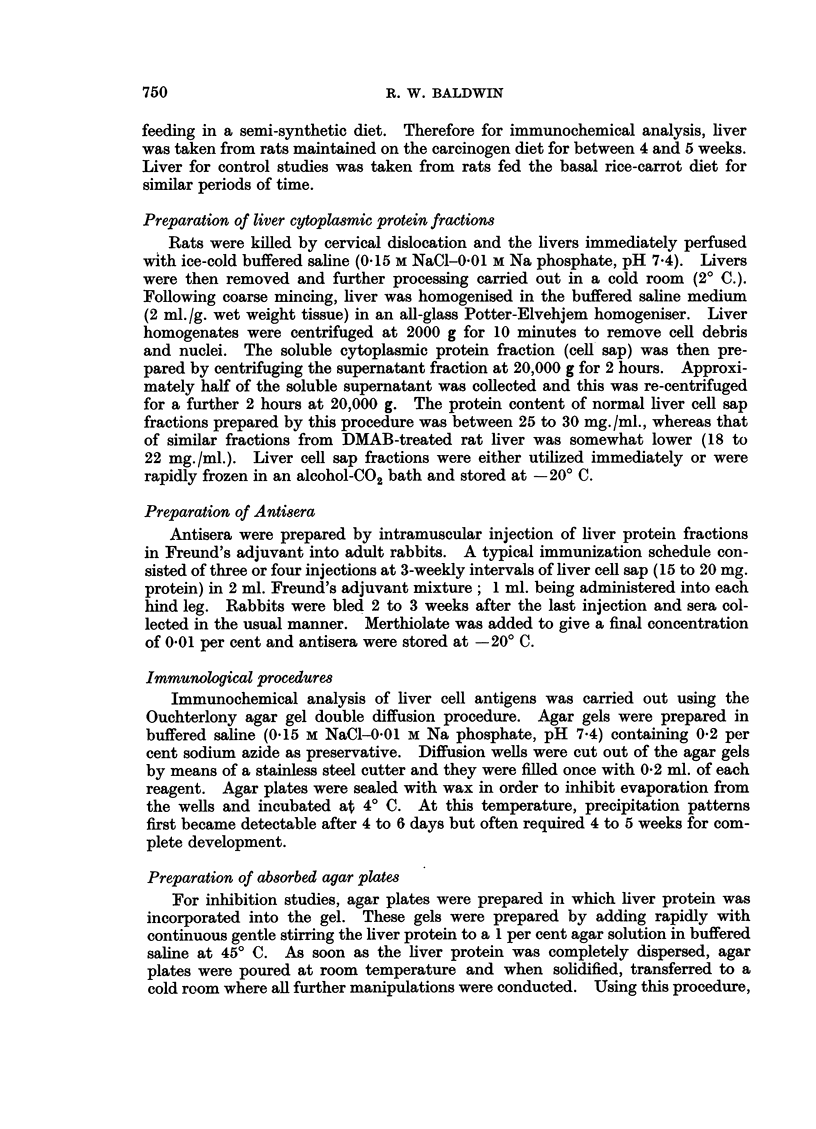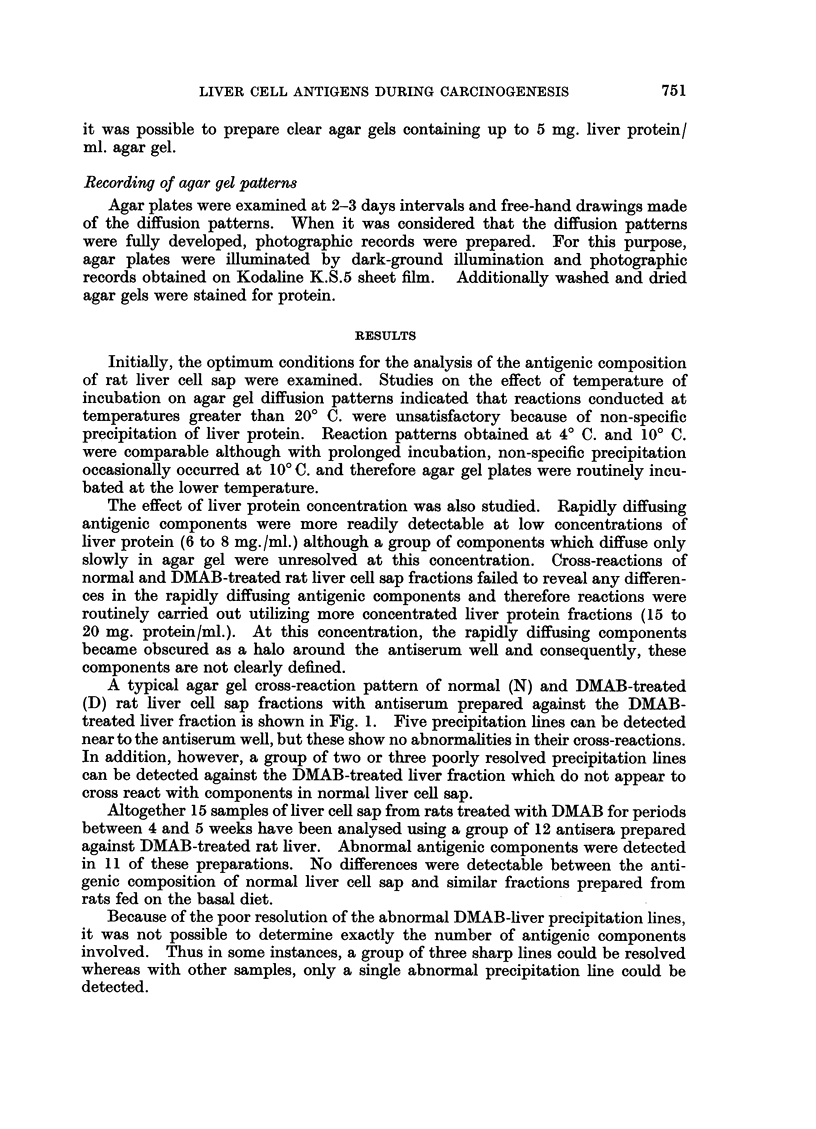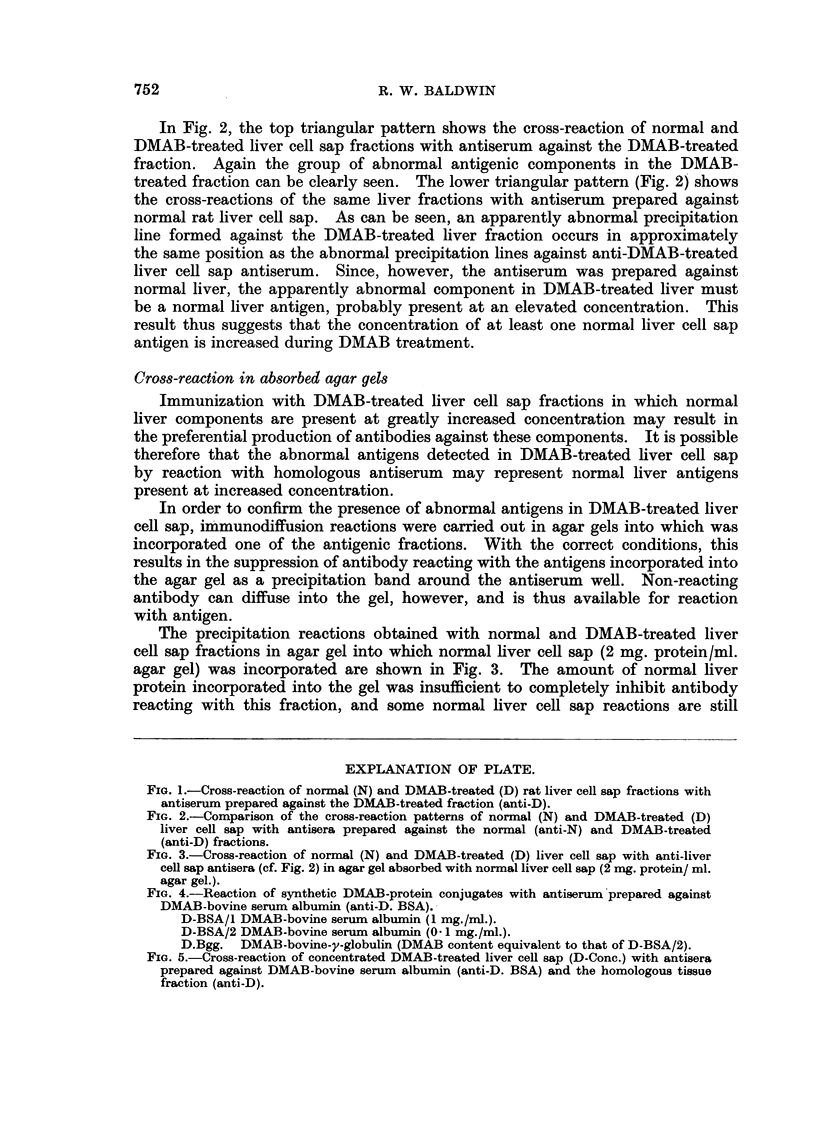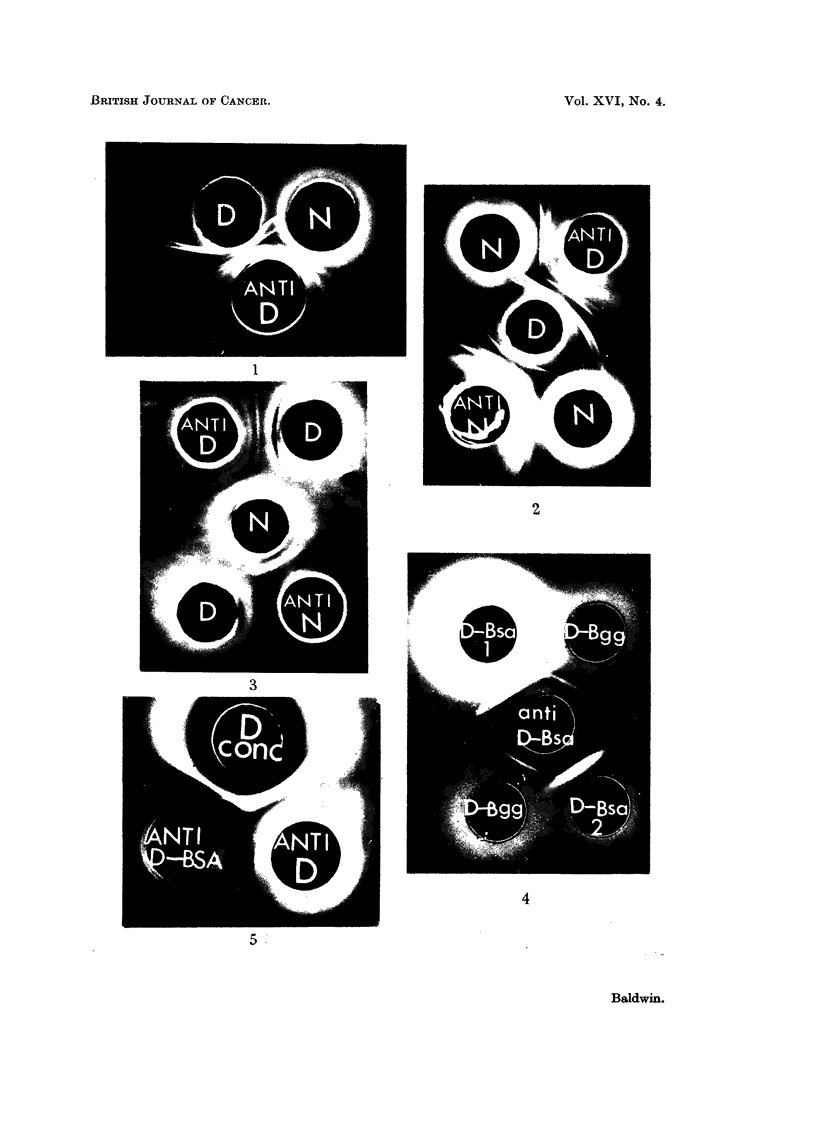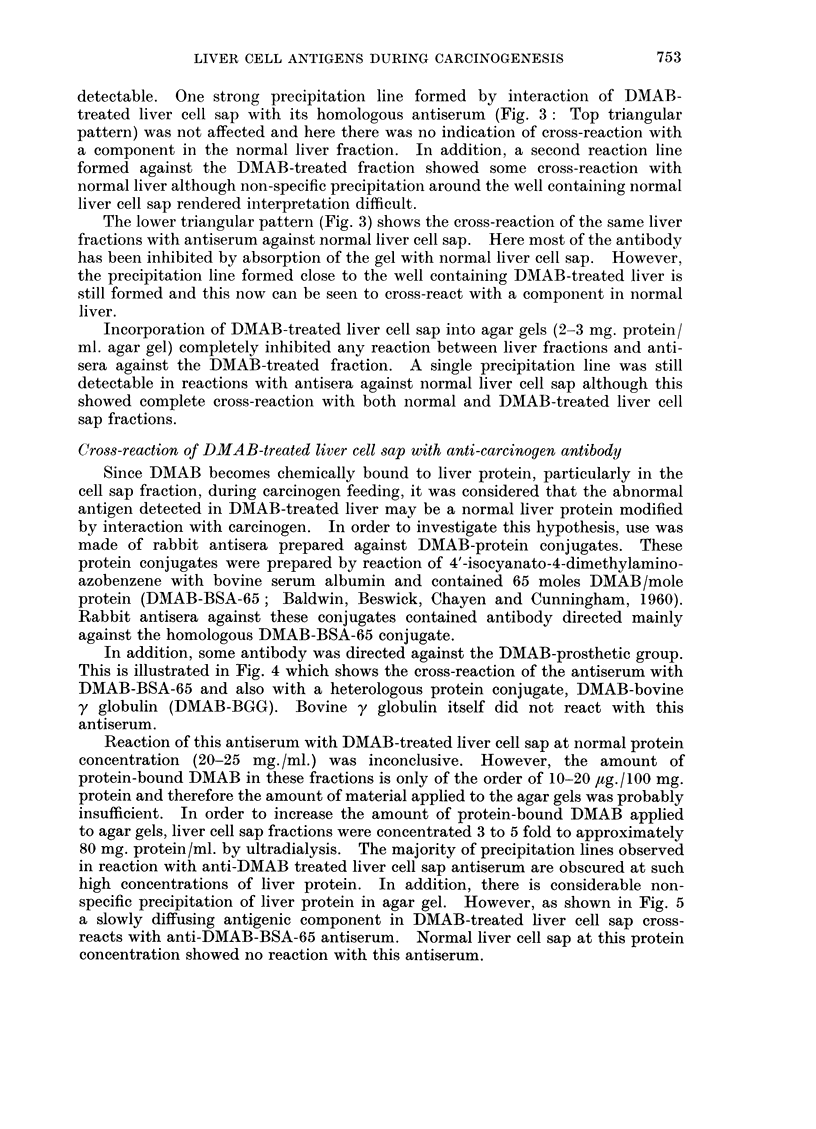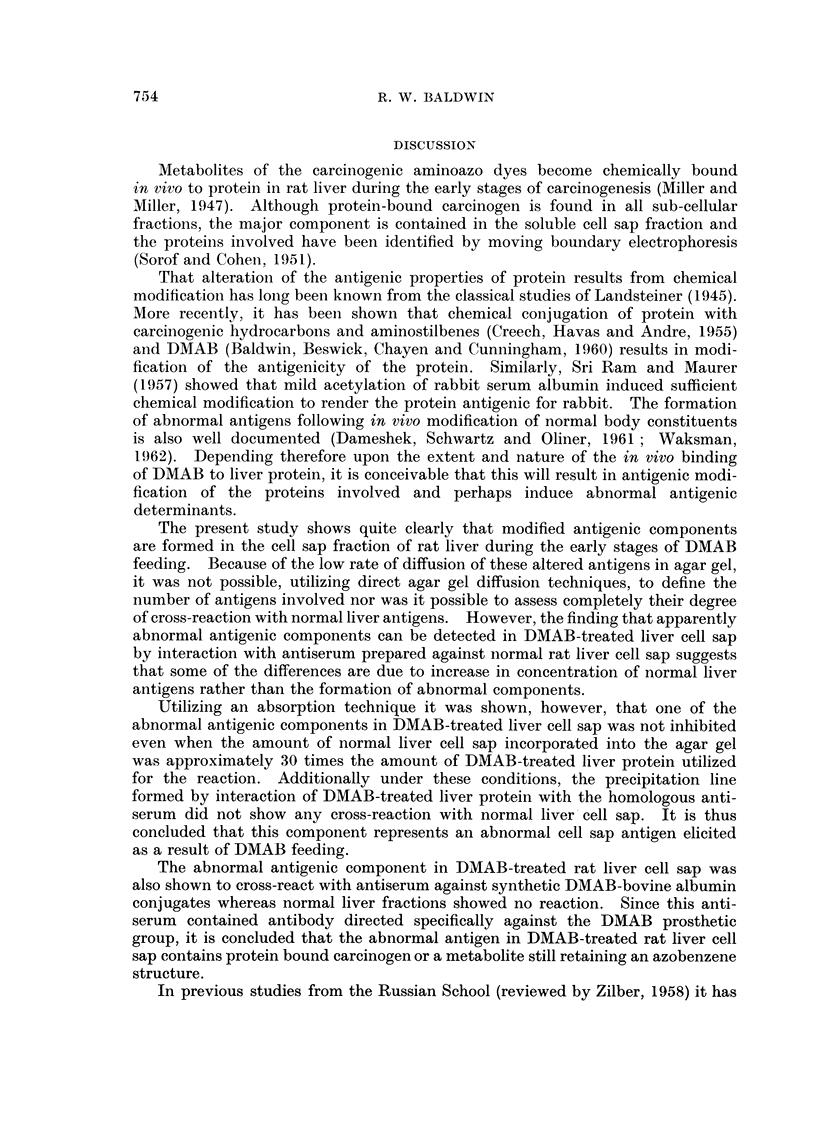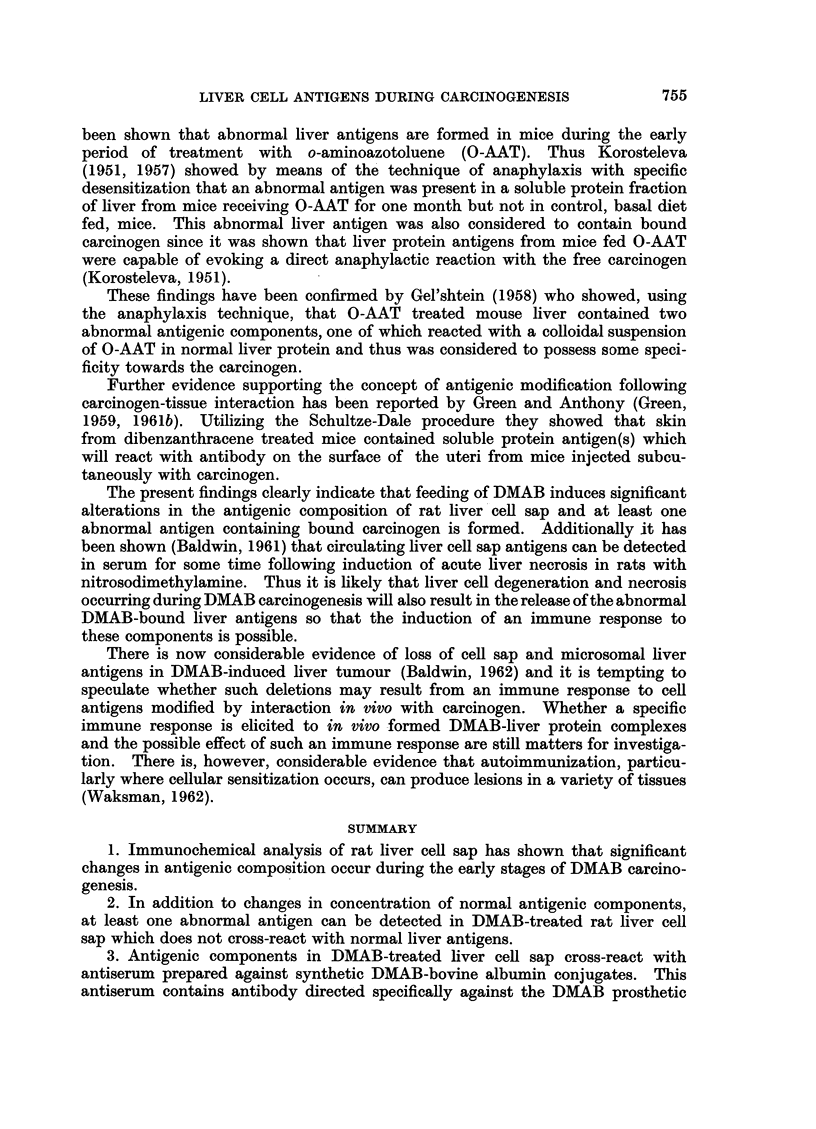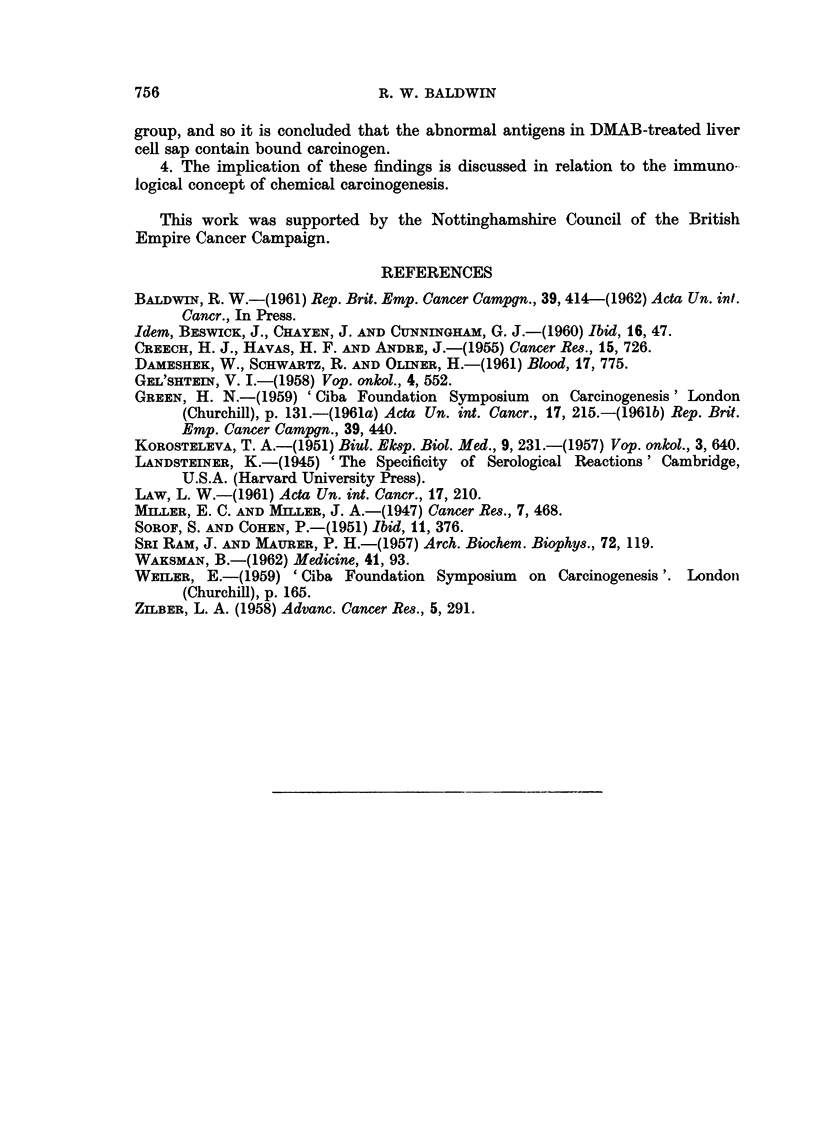# Studies on Rat Liver Cell Antigens During the Early Stages of Azo Dye Carcinogenesis

**DOI:** 10.1038/bjc.1962.87

**Published:** 1962-12

**Authors:** R. W. Baldwin

## Abstract

**Images:**


### STUDIES ON RAT LIVER CELL ANTIGENS DURING THE EARLY STAGES OF AZO DYE CARCINOGENESIS

R. W. BALDWIN

*From the Cancer Research Laboratory, The University, Nottingham*

Received for publication October 29, 1962

ATTENTION has been drawn in recent years to the immunological concept of the aetiology of carcinogenesis (Green, 1961 ; Law, 1961 ; Weiler, 1959). The essential feature of these hypotheses is that there is a loss of tissue specific antigens during carcinogenesis. According to Green (1961*a*), this deletion may result from adaptation to an immune response induced against antigenic complexes formed following interaction of carcinogen with tissue-specific antigens.

In order for such a concept to be meaningful, it is necessary to show that :

1. Modified cell antigens are formed during the early stages of chemical carcinogenesis.
2. These modified cell antigens can be recognised as " foreign " by the host and so induce an immune response.
3. There is a loss of cell antigens during the change from a normal to a malignant cell and that this loss results from adaptation to an immune response evoked by modified cell antigens.

The present paper describes a study of the formation of abnormal tissue antigens in rat liver during the early stages of 4-dimethylaminoazobenzene (DMAB) carcinogenesis. This system was selected for investigation since Miller and Miller (1947) showed that a metabolite of DMAB becomes chemically bound to liver protein, particularly in the soluble cell sap fraction, during the early stages of carcinogen feeding. The level of this protein-bound metabolite in liver reached a maximum after 4 to 6 weeks of carcinogen treatment and thereafter gradually decreased. Although the metabolite could still be detected in liver even after 20 weeks of carcinogen feeding, none was found in induced liver tumour. This inability of tumour to bind DMAB to protein and the concurrent loss of certain cytoplasmic proteins involved in carcinogen binding (Sorof and Cohen, 1951) has been interpreted as indicating qualitative differences between the proteins of normal liver and induced tumour.

### MATERIALS AND METHODS

*Normal rat liver* was taken from adult male Wistar rats maintained on a standard cubed diet with water *ad libitum.*

*DMAB-treated rat liver.*—Adult male Wistar rats (250–300 g.) were maintained on a rice-carrot diet containing 0·06 per cent DMAB. Estimation of the protein bound metabolite of DMAB by the procedure of Miller and Miller (1947) indicated that the maximal level of carcinogen binding occurred after 4 to 6 weeks feeding and was comparable to that reported by Miller and Miller (1947) following DMAB

feeding in a semi-synthetic diet. Therefore for immunochemical analysis, liver was taken from rats maintained on the carcinogen diet for between 4 and 5 weeks. Liver for control studies was taken from rats fed the basal rice-carrot diet for similar periods of time.

*Preparation of liver cytoplasmic protein fractions*

Rats were killed by cervical dislocation and the livers immediately perfused with ice-cold buffered saline (0·15 M NaCl–0·01 M Na phosphate, pH 7·4). Livers were then removed and further processing carried out in a cold room (2° C.). Following coarse mincing, liver was homogenised in the buffered saline medium (2 ml./g. wet weight tissue) in an all-glass Potter-Elvehjem homogeniser. Liver homogenates were centrifuged at 2000 **g** for 10 minutes to remove cell debris and nuclei. The soluble cytoplasmic protein fraction (cell sap) was then prepared by centrifuging the supernatant fraction at 20,000 **g** for 2 hours. Approximately half of the soluble supernatant was collected and this was re-centrifuged for a further 2 hours at 20,000 **g**. The protein content of normal liver cell sap fractions prepared by this procedure was between 25 to 30 mg./ml., whereas that of similar fractions from DMAB-treated rat liver was somewhat lower (18 to 22 mg./ml.). Liver cell sap fractions were either utilized immediately or were rapidly frozen in an alcohol-$CO_2$ bath and stored at $-20°$ C.

*Preparation of Antisera*

Antisera were prepared by intramuscular injection of liver protein fractions in Freund's adjuvant into adult rabbits. A typical immunization schedule consisted of three or four injections at 3-weekly intervals of liver cell sap (15 to 20 mg. protein) in 2 ml. Freund's adjuvant mixture ; 1 ml. being administered into each hind leg. Rabbits were bled 2 to 3 weeks after the last injection and sera collected in the usual manner. Merthiolate was added to give a final concentration of 0·01 per cent and antisera were stored at $-20°$ C.

*Immunological procedures*

Immunochemical analysis of liver cell antigens was carried out using the Ouchterlony agar gel double diffusion procedure. Agar gels were prepared in buffered saline (0·15 M NaCl–0·01 M Na phosphate, pH 7·4) containing 0·2 per cent sodium azide as preservative. Diffusion wells were cut out of the agar gels by means of a stainless steel cutter and they were filled once with 0·2 ml. of each reagent. Agar plates were sealed with wax in order to inhibit evaporation from the wells and incubated at 4° C. At this temperature, precipitation patterns first became detectable after 4 to 6 days but often required 4 to 5 weeks for complete development.

*Preparation of absorbed agar plates*

For inhibition studies, agar plates were prepared in which liver protein was incorporated into the gel. These gels were prepared by adding rapidly with continuous gentle stirring the liver protein to a 1 per cent agar solution in buffered saline at 45° C. As soon as the liver protein was completely dispersed, agar plates were poured at room temperature and when solidified, transferred to a cold room where all further manipulations were conducted. Using this procedure, it was possible to prepare clear agar gels containing up to 5 mg. liver protein/ml. agar gel.

*Recording of agar gel patterns*

Agar plates were examined at 2–3 days intervals and free-hand drawings made of the diffusion patterns. When it was considered that the diffusion patterns were fully developed, photographic records were prepared. For this purpose, agar plates were illuminated by dark-ground illumination and photographic records obtained on Kodaline K.S.5 sheet film. Additionally washed and dried agar gels were stained for protein.

### RESULTS

Initially, the optimum conditions for the analysis of the antigenic composition of rat liver cell sap were examined. Studies on the effect of temperature of incubation on agar gel diffusion patterns indicated that reactions conducted at temperatures greater than 20° C. were unsatisfactory because of non-specific precipitation of liver protein. Reaction patterns obtained at 4° C. and 10° C. were comparable although with prolonged incubation, non-specific precipitation occasionally occurred at 10° C. and therefore agar gel plates were routinely incubated at the lower temperature.

The effect of liver protein concentration was also studied. Rapidly diffusing antigenic components were more readily detectable at low concentrations of liver protein (6 to 8 mg./ml.) although a group of components which diffuse only slowly in agar gel were unresolved at this concentration. Cross-reactions of normal and DMAB-treated rat liver cell sap fractions failed to reveal any differences in the rapidly diffusing antigenic components and therefore reactions were routinely carried out utilizing more concentrated liver protein fractions (15 to 20 mg. protein/ml.). At this concentration, the rapidly diffusing components became obscured as a halo around the antiserum well and consequently, these components are not clearly defined.

A typical agar gel cross-reaction pattern of normal (N) and DMAB-treated (D) rat liver cell sap fractions with antiserum prepared against the DMAB-treated liver fraction is shown in Fig. 1. Five precipitation lines can be detected near to the antiserum well, but these show no abnormalities in their cross-reactions. In addition, however, a group of two or three poorly resolved precipitation lines can be detected against the DMAB-treated liver fraction which do not appear to cross react with components in normal liver cell sap.

Altogether 15 samples of liver cell sap from rats treated with DMAB for periods between 4 and 5 weeks have been analysed using a group of 12 antisera prepared against DMAB-treated rat liver. Abnormal antigenic components were detected in 11 of these preparations. No differences were detectable between the antigenic composition of normal liver cell sap and similar fractions prepared from rats fed on the basal diet.

Because of the poor resolution of the abnormal DMAB-liver precipitation lines, it was not possible to determine exactly the number of antigenic components involved. Thus in some instances, a group of three sharp lines could be resolved whereas with other samples, only a single abnormal precipitation line could be detected.

In Fig. 2, the top triangular pattern shows the cross-reaction of normal and DMAB-treated liver cell sap fractions with antiserum against the DMAB-treated fraction. Again the group of abnormal antigenic components in the DMAB-treated fraction can be clearly seen. The lower triangular pattern (Fig. 2) shows the cross-reactions of the same liver fractions with antiserum prepared against normal rat liver cell sap. As can be seen, an apparently abnormal precipitation line formed against the DMAB-treated liver fraction occurs in approximately the same position as the abnormal precipitation lines against anti-DMAB-treated liver cell sap antiserum. Since, however, the antiserum was prepared against normal liver, the apparently abnormal component in DMAB-treated liver must be a normal liver antigen, probably present at an elevated concentration. This result thus suggests that the concentration of at least one normal liver cell sap antigen is increased during DMAB treatment.

*Cross-reaction in absorbed agar gels*

Immunization with DMAB-treated liver cell sap fractions in which normal liver components are present at greatly increased concentration may result in the preferential production of antibodies against these components. It is possible therefore that the abnormal antigens detected in DMAB-treated liver cell sap by reaction with homologous antiserum may represent normal liver antigens present at increased concentration.

In order to confirm the presence of abnormal antigens in DMAB-treated liver cell sap, immunodiffusion reactions were carried out in agar gels into which was incorporated one of the antigenic fractions. With the correct conditions, this results in the suppression of antibody reacting with the antigens incorporated into the agar gel as a precipitation band around the antiserum well. Non-reacting antibody can diffuse into the gel, however, and is thus available for reaction with antigen.

The precipitation reactions obtained with normal and DMAB-treated liver cell sap fractions in agar gel into which normal liver cell sap (2 mg. protein/ml. agar gel) was incorporated are shown in Fig. 3. The amount of normal liver protein incorporated into the gel was insufficient to completely inhibit antibody reacting with this fraction, and some normal liver cell sap reactions are still

---

EXPLANATION OF PLATE.

FIG. 1.—Cross-reaction of normal (N) and DMAB-treated (D) rat liver cell sap fractions with antiserum prepared against the DMAB-treated fraction (anti-D).

FIG. 2.—Comparison of the cross-reaction patterns of normal (N) and DMAB-treated (D) liver cell sap with antisera prepared against the normal (anti-N) and DMAB-treated (anti-D) fractions.

FIG. 3.—Cross-reaction of normal (N) and DMAB-treated (D) liver cell sap with anti-liver cell sap antisera (cf. Fig. 2) in agar gel absorbed with normal liver cell sap (2 mg. protein/ ml. agar gel.).

FIG. 4.—Reaction of synthetic DMAB-protein conjugates with antiserum prepared against DMAB-bovine serum albumin (anti-D. BSA).

D-BSA/1 DMAB-bovine serum albumin (1 mg./ml.).

D-BSA/2 DMAB-bovine serum albumin (0·1 mg./ml.).

D.Bgg. DMAB-bovine-$\gamma$-globulin (DMAB content equivalent to that of D-BSA/2).

FIG. 5.—Cross-reaction of concentrated DMAB-treated liver cell sap (D-Conc.) with antisera prepared against DMAB-bovine serum albumin (anti-D. BSA) and the homologous tissue fraction (anti-D).

1

2

3

4

5

Baldwin.

detectable. One strong precipitation line formed by interaction of DMAB-treated liver cell sap with its homologous antiserum (Fig. 3 : Top triangular pattern) was not affected and here there was no indication of cross-reaction with a component in the normal liver fraction. In addition, a second reaction line formed against the DMAB-treated fraction showed some cross-reaction with normal liver although non-specific precipitation around the well containing normal liver cell sap rendered interpretation difficult.

The lower triangular pattern (Fig. 3) shows the cross-reaction of the same liver fractions with antiserum against normal liver cell sap. Here most of the antibody has been inhibited by absorption of the gel with normal liver cell sap. However, the precipitation line formed close to the well containing DMAB-treated liver is still formed and this now can be seen to cross-react with a component in normal liver.

Incorporation of DMAB-treated liver cell sap into agar gels (2–3 mg. protein/ml. agar gel) completely inhibited any reaction between liver fractions and antisera against the DMAB-treated fraction. A single precipitation line was still detectable in reactions with antisera against normal liver cell sap although this showed complete cross-reaction with both normal and DMAB-treated liver cell sap fractions.

*Cross-reaction of DMAB-treated liver cell sap with anti-carcinogen antibody*

Since DMAB becomes chemically bound to liver protein, particularly in the cell sap fraction, during carcinogen feeding, it was considered that the abnormal antigen detected in DMAB-treated liver may be a normal liver protein modified by interaction with carcinogen. In order to investigate this hypothesis, use was made of rabbit antisera prepared against DMAB-protein conjugates. These protein conjugates were prepared by reaction of 4′-isocyanato-4-dimethylamino-azobenzene with bovine serum albumin and contained 65 moles DMAB/mole protein (DMAB-BSA-65 ; Baldwin, Beswick, Chayen and Cunningham, 1960). Rabbit antisera against these conjugates contained antibody directed mainly against the homologous DMAB-BSA-65 conjugate.

In addition, some antibody was directed against the DMAB-prosthetic group. This is illustrated in Fig. 4 which shows the cross-reaction of the antiserum with DMAB-BSA-65 and also with a heterologous protein conjugate, DMAB-bovine $\gamma$ globulin (DMAB-BGG). Bovine $\gamma$ globulin itself did not react with this antiserum.

Reaction of this antiserum with DMAB-treated liver cell sap at normal protein concentration (20–25 mg./ml.) was inconclusive. However, the amount of protein-bound DMAB in these fractions is only of the order of 10–20 $\mu$g./100 mg. protein and therefore the amount of material applied to the agar gels was probably insufficient. In order to increase the amount of protein-bound DMAB applied to agar gels, liver cell sap fractions were concentrated 3 to 5 fold to approximately 80 mg. protein/ml. by ultradialysis. The majority of precipitation lines observed in reaction with anti-DMAB treated liver cell sap antiserum are obscured at such high concentrations of liver protein. In addition, there is considerable non-specific precipitation of liver protein in agar gel. However, as shown in Fig. 5 a slowly diffusing antigenic component in DMAB-treated liver cell sap cross-reacts with anti-DMAB-BSA-65 antiserum. Normal liver cell sap at this protein concentration showed no reaction with this antiserum.

### DISCUSSION

Metabolites of the carcinogenic aminoazo dyes become chemically bound *in vivo* to protein in rat liver during the early stages of carcinogenesis (Miller and Miller, 1947). Although protein-bound carcinogen is found in all sub-cellular fractions, the major component is contained in the soluble cell sap fraction and the proteins involved have been identified by moving boundary electrophoresis (Sorof and Cohen, 1951).

That alteration of the antigenic properties of protein results from chemical modification has long been known from the classical studies of Landsteiner (1945). More recently, it has been shown that chemical conjugation of protein with carcinogenic hydrocarbons and aminostilbenes (Creech, Havas and Andre, 1955) and DMAB (Baldwin, Beswick, Chayen and Cunningham, 1960) results in modification of the antigenicity of the protein. Similarly, Sri Ram and Maurer (1957) showed that mild acetylation of rabbit serum albumin induced sufficient chemical modification to render the protein antigenic for rabbit. The formation of abnormal antigens following *in vivo* modification of normal body constituents is also well documented (Dameshek, Schwartz and Oliner, 1961 ; Waksman, 1962). Depending therefore upon the extent and nature of the *in vivo* binding of DMAB to liver protein, it is conceivable that this will result in antigenic modification of the proteins involved and perhaps induce abnormal antigenic determinants.

The present study shows quite clearly that modified antigenic components are formed in the cell sap fraction of rat liver during the early stages of DMAB feeding. Because of the low rate of diffusion of these altered antigens in agar gel, it was not possible, utilizing direct agar gel diffusion techniques, to define the number of antigens involved nor was it possible to assess completely their degree of cross-reaction with normal liver antigens. However, the finding that apparently abnormal antigenic components can be detected in DMAB-treated liver cell sap by interaction with antiserum prepared against normal rat liver cell sap suggests that some of the differences are due to increase in concentration of normal liver antigens rather than the formation of abnormal components.

Utilizing an absorption technique it was shown, however, that one of the abnormal antigenic components in DMAB-treated liver cell sap was not inhibited even when the amount of normal liver cell sap incorporated into the agar gel was approximately 30 times the amount of DMAB-treated liver protein utilized for the reaction. Additionally under these conditions, the precipitation line formed by interaction of DMAB-treated liver protein with the homologous antiserum did not show any cross-reaction with normal liver cell sap. It is thus concluded that this component represents an abnormal cell sap antigen elicited as a result of DMAB feeding.

The abnormal antigenic component in DMAB-treated rat liver cell sap was also shown to cross-react with antiserum against synthetic DMAB-bovine albumin conjugates whereas normal liver fractions showed no reaction. Since this antiserum contained antibody directed specifically against the DMAB prosthetic group, it is concluded that the abnormal antigen in DMAB-treated rat liver cell sap contains protein bound carcinogen or a metabolite still retaining an azobenzene structure.

In previous studies from the Russian School (reviewed by Zilber, 1958) it has been shown that abnormal liver antigens are formed in mice during the early period of treatment with *o*-aminoazotoluene (O-AAT). Thus Korosteleva (1951, 1957) showed by means of the technique of anaphylaxis with specific desensitization that an abnormal antigen was present in a soluble protein fraction of liver from mice receiving O-AAT for one month but not in control, basal diet fed, mice. This abnormal liver antigen was also considered to contain bound carcinogen since it was shown that liver protein antigens from mice fed O-AAT were capable of evoking a direct anaphylactic reaction with the free carcinogen (Korosteleva, 1951).

These findings have been confirmed by Gel'shtein (1958) who showed, using the anaphylaxis technique, that O-AAT treated mouse liver contained two abnormal antigenic components, one of which reacted with a colloidal suspension of O-AAT in normal liver protein and thus was considered to possess some specificity towards the carcinogen.

Further evidence supporting the concept of antigenic modification following carcinogen-tissue interaction has been reported by Green and Anthony (Green, 1959, 1961*b*). Utilizing the Schultze-Dale procedure they showed that skin from dibenzanthracene treated mice contained soluble protein antigen(s) which will react with antibody on the surface of the uteri from mice injected subcutaneously with carcinogen.

The present findings clearly indicate that feeding of DMAB induces significant alterations in the antigenic composition of rat liver cell sap and at least one abnormal antigen containing bound carcinogen is formed. Additionally it has been shown (Baldwin, 1961) that circulating liver cell sap antigens can be detected in serum for some time following induction of acute liver necrosis in rats with nitrosodimethylamine. Thus it is likely that liver cell degeneration and necrosis occurring during DMAB carcinogenesis will also result in the release of the abnormal DMAB-bound liver antigens so that the induction of an immune response to these components is possible.

There is now considerable evidence of loss of cell sap and microsomal liver antigens in DMAB-induced liver tumour (Baldwin, 1962) and it is tempting to speculate whether such deletions may result from an immune response to cell antigens modified by interaction *in vivo* with carcinogen. Whether a specific immune response is elicited to *in vivo* formed DMAB-liver protein complexes and the possible effect of such an immune response are still matters for investigation. There is, however, considerable evidence that autoimmunization, particularly where cellular sensitization occurs, can produce lesions in a variety of tissues (Waksman, 1962).

### SUMMARY

1. Immunochemical analysis of rat liver cell sap has shown that significant changes in antigenic composition occur during the early stages of DMAB carcinogenesis.

2. In addition to changes in concentration of normal antigenic components, at least one abnormal antigen can be detected in DMAB-treated rat liver cell sap which does not cross-react with normal liver antigens.

3. Antigenic components in DMAB-treated liver cell sap cross-react with antiserum prepared against synthetic DMAB-bovine albumin conjugates. This antiserum contains antibody directed specifically against the DMAB prosthetic group, and so it is concluded that the abnormal antigens in DMAB-treated liver cell sap contain bound carcinogen.

4. The implication of these findings is discussed in relation to the immunological concept of chemical carcinogenesis.

This work was supported by the Nottinghamshire Council of the British Empire Cancer Campaign.

### REFERENCES

BALDWIN, R. W.—(1961) *Rep. Brit. Emp. Cancer Campgn.*, **39**, 414—(1962) *Acta Un. int. Cancr.*, In Press.

*Idem*, BESWICK, J., CHAYEN, J. AND CUNNINGHAM, G. J.—(1960) *Ibid*, **16**, 47.

CREECH, H. J., HAVAS, H. F. AND ANDRE, J.—(1955) *Cancer Res.*, **15**, 726.

DAMESHEK, W., SCHWARTZ, R. AND OLINER, H.—(1961) *Blood*, **17**, 775.

GEL'SHTEIN, V. I.—(1958) *Vop. onkol.*, **4**, 552.

GREEN, H. N.—(1959) 'Ciba Foundation Symposium on Carcinogenesis' London (Churchill), p. 131.—(1961*a*) *Acta Un. int. Cancr.*, **17**, 215.—(1961*b*) *Rep. Brit. Emp. Cancer Campgn.*, **39**, 440.

KOROSTELEVA, T. A.—(1951) *Biul. Eksp. Biol. Med.*, **9**, 231.—(1957) *Vop. onkol.*, **3**, 640.

LANDSTEINER, K.—(1945) 'The Specificity of Serological Reactions' Cambridge, U.S.A. (Harvard University Press).

LAW, L. W.—(1961) *Acta Un. int. Cancr.*, **17**, 210.

MILLER, E. C. AND MILLER, J. A.—(1947) *Cancer Res.*, **7**, 468.

SOROF, S. AND COHEN, P.—(1951) *Ibid*, **11**, 376.

SRI RAM, J. AND MAURER, P. H.—(1957) *Arch. Biochem. Biophys.*, **72**, 119.

WAKSMAN, B.—(1962) *Medicine*, **41**, 93.

WEILER, E.—(1959) 'Ciba Foundation Symposium on Carcinogenesis'. London (Churchill), p. 165.

ZILBER, L. A. (1958) *Advanc. Cancer Res.*, **5**, 291.